# Supplementary material for: Detecting latent interaction effects when analyzing binary traits
Source: PLoS Genet. 2025 Aug 22;21(8):e1011822. doi: 10.1371/journal.pgen.1011822 (PMC12396767; doi:10.1371/journal.pgen.1011822)
Supplement: S1 Table — However, as explained in the paper, there will be no model-misspecification issue even when the original model is not probit. The notation p denotes the minor allele frequency and q = 1−p. (PDF) [file pgen.1011822.s001.pdf]

Model	Specification	Parameters
Original model	$\beta_0 + \beta_G G_A + \beta_E E + \beta_{GE} G_A E$<br>$G_A = (0,1,2)$ for (aa, Aa, AA)	-
Saturated model	$\gamma_0 I(G = aa) + \gamma_1 I(G = Aa) + \gamma_2 I(G = AA)$	$\gamma_0 = \frac{\beta_0}{\beta_E^2 \sigma_E^2 + 1}$,<br>$\gamma_1 = \frac{\beta_0 + \beta_G}{(\beta_E + \beta_{GE})^2 \sigma_E^2 + 1}$,<br>$\gamma_2 = \frac{\beta_0 + 2\beta_G}{(\beta_E + 2\beta_{GE})^2 \sigma_E^2 + 1}$,<br>$\gamma_D = \gamma_2 - 2\gamma_1 + \gamma_0$<br>(if $\beta_{GE} = 0$ then $\gamma_D = 0$).
Orthogonal coding	$\beta_0^* + \beta_A^* G_A + \beta_D^* G_D$<br>$G_A = (0,1,2)$ for (aa, Aa, AA)<br>$G_D = (-p/q, 1, -q/p)$ for (aa, Aa, AA)	$\beta_0^* = (1 - p^2)\gamma_0 + p^2(2\gamma_1 - \gamma_2)$,<br>$\beta_A^* = -q\gamma_0 - (p - q)\gamma_1 + p\gamma_2$<br>$= p\gamma_D + (\gamma_1 - \gamma_0)$,<br>(if $\beta_{GE} = 0$ then $\beta_A^* = \beta_G$).<br>$\beta_D^* = -pq\gamma_D$

Table S1: Different parameterization of the saturated model: The parameters parameterization of $\gamma_0, \gamma_1, \gamma_2$ in terms of $\beta_0, \beta_G, \beta_{GE}$ assumes that the original model is probit. However, as explained in the paper, there will be no model-misspecification issue even when the original model is not probit. The notation p denotes the minor allele frequency and $q = 1 - p$.
