# Supplementary material for: Detecting latent interaction effects when analyzing binary traits
Source: PLoS Genet. 2025 Aug 22;21(8):e1011822. doi: 10.1371/journal.pgen.1011822 (PMC12396767; doi:10.1371/journal.pgen.1011822)
Supplement: S2 Table — (PDF) [file pgen.1011822.s002.pdf]

Table S2: Summary of characteristics of all the 65 SNPs studied in the interaction analysis; including minor allele frequencies, linkage disequilibrium measures (D' and R^2), distance (in BP) to rs7412, and association test p-values (Indirect and Interaction) along with estimated regression coefficients ($\hat{\beta}_D$ and $\hat{\beta}_{GE}$).

	SNP	MAF	D'	R^2	Distance	p: Indirect	p: Interaction	$\hat{\beta}_D$	$\hat{\beta}_{GE}$
1	rs7254892	0.03	0.93	0.41	-22483	3.723e-03	1.713e-07	-1.843e-02	4.826e-02
2	rs141622900	0.04	0.95	0.64	14713	6.685e-14	1.960e-07	-4.742e-02	6.425e-02
3	rs34954997	0.22	1.00	0.24	5559	3.402e-01	2.733e-07	-6.002e-03	5.764e-02
4	rs483082	0.22	1.00	0.24	4099	3.166e-01	4.940e-07	-6.291e-03	5.684e-02
5	rs405509	0.48	1.00	0.06	-3243	3.849e-01	3.467e-06	-5.203e-03	-4.932e-02
6	rs440446	0.36	1.00	0.04	-2912	9.960e-01	4.849e-06	-3.244e-05	-4.909e-02
7	rs75627662	0.19	1.00	0.29	1497	3.514e-02	6.094e-06	-1.337e-02	5.253e-02
8	rs72654473	0.09	1.00	0.65	2320	3.613e-10	1.337e-05	-4.249e-02	6.595e-02
9	rs439401	0.38	1.00	0.04	2372	6.640e-01	2.835e-05	2.638e-03	-4.502e-02
10	rs584007	0.38	1.00	0.04	4399	8.491e-01	8.011e-05	-1.235e-03	-4.235e-02
11	rs445925	0.09	1.00	0.64	3561	1.593e-11	1.825e-04	-4.191e-02	6.027e-02
12	rs62120566	0.01	0.43	0.04	-214347	2.741e-01	2.564e-03	-7.277e-03	1.535e-02
13	rs7259004	0.10	0.51	0.16	20478	6.523e-01	3.433e-03	-2.774e-03	1.706e-02
14	rs405697	0.27	1.00	0.02	-7388	6.944e-01	3.688e-03	2.566e-03	-3.079e-02
15	rs62119327	0.01	0.29	0.02	-225206	1.582e-01	8.136e-03	-9.523e-03	1.322e-02
16	rs2965169	0.44	0.63	0.03	-160923	3.917e-01	1.229e-02	-5.213e-03	2.014e-02
17	rs184017	0.21	0.36	0.03	-17110	3.901e-01	1.476e-02	5.214e-03	-1.636e-02
18	rs283815	0.21	0.36	0.03	-21746	3.202e-01	1.788e-02	6.073e-03	-1.593e-02
19	rs59007384	0.21	0.36	0.03	-15414	1.944e-01	1.867e-02	7.893e-03	-1.566e-02
20	rs157581	0.21	0.36	0.03	-16365	5.273e-01	2.259e-02	3.600e-03	-1.525e-02
21	rs76560105	0.02	0.47	0.08	-112880	1.745e-04	2.285e-02	-2.179e-02	1.148e-02
22	rs157582	0.21	0.36	0.03	-15860	3.239e-01	2.386e-02	6.004e-03	-1.511e-02
23	rs28399637	0.32	0.66	0.01	-87941	5.845e-02	2.708e-02	1.117e-02	-2.074e-02
24	rs11668861	0.46	0.79	0.03	-31109	4.978e-01	2.890e-02	-4.451e-03	-2.138e-02
25	rs28399654	0.03	0.45	0.09	-95491	7.565e-04	7.091e-02	-1.964e-02	9.116e-03
26	rs11668327	0.16	0.42	0.06	-13446	2.293e-02	7.287e-02	1.583e-02	1.218e-02
27	rs10411821	0.33	0.29	0.01	684053	6.518e-01	1.679e-01	2.713e-03	1.002e-02
28	rs519113	0.21	0.33	0.03	-35795	4.205e-01	2.099e-01	-4.921e-03	8.759e-03
29	rs2965101	0.35	0.39	0.02	-174267	1.031e-01	2.162e-01	-9.945e-03	8.969e-03
30	rs8109620	0.31	0.40	0.02	250380	3.968e-01	2.601e-01	-5.087e-03	-8.093e-03
31	rs10405693	0.34	0.72	0.02	-85415	8.591e-01	2.620e-01	-1.044e-03	-1.080e-02
32	rs6859	0.44	0.82	0.04	-30045	5.918e-01	2.631e-01	-3.184e-03	-1.053e-02

Continued on next page

Table S2 continued from previous page

	SNP	MAF	D'	R^2	Distance	p: Indirect	p: Interaction	$\hat{\beta}_D$	$\hat{\beta}_{GE}$
33	rs1531517	0.05	0.36	0.11	-169906	8.796e-01	2.647e-01	-1.010e-03	6.161e-03
34	rs429358	0.16	1.00	0.01	-138	1.988e-01	2.842e-01	7.619e-03	-1.079e-02
35	rs4420638	0.20	0.92	0.01	10867	3.210e-01	2.927e-01	5.483e-03	-1.025e-02
36	rs365653	0.09	0.37	0.09	-50433	5.045e-01	3.009e-01	-4.677e-03	6.320e-03
37	rs28399653	0.03	0.45	0.09	-96634	5.861e-02	3.257e-01	-1.153e-02	5.109e-03
38	rs12721109	0.02	0.76	0.16	35142	3.646e-02	3.660e-01	-1.327e-02	-5.204e-03
39	rs8104483	0.29	0.73	0.01	-39725	5.929e-01	3.955e-01	3.165e-03	-7.920e-03
40	rs283813	0.08	0.42	0.14	-22905	1.689e-01	4.030e-01	-8.716e-03	-4.444e-03
41	rs11672271	0.31	0.42	0.03	207867	7.355e-01	4.150e-01	-2.030e-03	-5.871e-03
42	rs16979890	0.14	0.20	0.02	654239	7.465e-01	5.052e-01	1.953e-03	4.656e-03
43	rs4803770	0.36	1.00	0.04	15274	9.462e-01	5.259e-01	4.303e-04	-6.574e-03
44	rs8106922	0.41	1.00	0.05	-10413	8.845e-01	5.262e-01	9.294e-04	6.321e-03
45	rs71352239	0.30	0.95	0.03	17464	9.858e-01	5.586e-01	-1.125e-04	5.636e-03
46	rs12162222	0.30	0.63	0.01	-63557	5.451e-01	5.789e-01	-3.543e-03	-5.352e-03
47	rs62117160	0.03	0.71	0.20	-179918	2.440e-01	5.993e-01	-7.985e-03	2.973e-03
48	rs3852856	0.21	0.78	0.01	-50505	9.406e-01	6.040e-01	4.713e-04	-5.208e-03
49	rs16979873	0.14	0.20	0.02	622432	9.010e-01	6.164e-01	-7.517e-04	-3.506e-03
50	rs35891370	0.35	0.29	0.01	107021	9.746e-01	6.499e-01	1.908e-04	-3.283e-03
51	rs59325138	0.40	1.00	0.04	4212	6.269e-01	6.530e-01	3.101e-03	-4.655e-03
52	rs769450	0.41	1.00	0.05	-1635	6.064e-01	7.116e-01	3.079e-03	-3.888e-03
53	rs3745150	0.44	0.93	0.04	-26320	5.988e-01	7.711e-01	-3.358e-03	-2.973e-03
54	rs3178166	0.46	0.41	0.01	182091	2.892e-01	7.807e-01	6.357e-03	-2.088e-03
55	rs421812	0.29	0.78	0.02	-31534	6.864e-01	7.920e-01	-2.603e-03	2.480e-03
56	rs45564734	0.02	0.26	0.02	-904898	3.584e-01	8.192e-01	-4.986e-03	-1.551e-03
57	rs73045960	0.02	0.37	0.04	883144	3.692e-02	8.684e-01	-1.062e-02	-8.778e-04
58	rs11881756	0.10	0.22	0.03	-191183	4.018e-01	8.686e-01	-5.260e-03	-9.878e-04
59	rs34978331	0.23	0.24	0.01	-79238	2.397e-01	8.939e-01	7.161e-03	9.333e-04
60	rs385982	0.33	0.76	0.02	-32397	6.673e-01	8.981e-01	-2.768e-03	-1.212e-03
61	rs369599	0.29	0.78	0.02	-32743	5.606e-01	9.186e-01	-3.742e-03	-9.678e-04
62	rs3837923	0.29	0.78	0.02	-32513	5.151e-01	9.483e-01	-4.184e-03	-6.124e-04
63	rs419925	0.29	0.78	0.02	-31953	5.128e-01	9.761e-01	-4.210e-03	-2.839e-04
64	rs2075649	0.42	0.96	0.04	-16749	5.640e-01	9.829e-01	-3.684e-03	-2.228e-04
65	rs370705	0.29	0.78	0.02	-32441	4.952e-01	9.952e-01	-4.385e-03	5.711e-05
61	rs369599	0.29	0.78	0.02	-32743	0.56	0.92	-0.00	-0.00
62	rs3837923	0.29	0.78	0.02	-32513	0.52	0.95	-0.00	-0.00

Continued on next page

Table S2 continued from previous page

	SNP	MAF	D'	R^2	Distance	p: Indirect	p: Interaction	$\hat{\beta}_D$	$\hat{\beta}_{GE}$
63	rs419925	0.29	0.78	0.02	-31953	0.51	0.98	-0.00	-0.00
64	rs2075649	0.42	0.96	0.04	-16749	0.56	0.98	-0.00	-0.00
65	rs370705	0.29	0.78	0.02	-32441	0.50	1.00	-0.00	0.00
