# Supplementary material for: Detecting latent interaction effects when analyzing binary traits
Source: PLoS Genet. 2025 Aug 22;21(8):e1011822. doi: 10.1371/journal.pgen.1011822 (PMC12396767; doi:10.1371/journal.pgen.1011822)
Supplement: S1 Fig — The parameter βE=0.5 and E∼N(0,1). The MAF is set to p = 0.3 in (C-D). The underlying model is assumed to be probit. The prevalence of the binary trait Y is 0.1 on the left column and 0.3 on the right column. (PDF) [file pgen.1011822.s003.pdf]

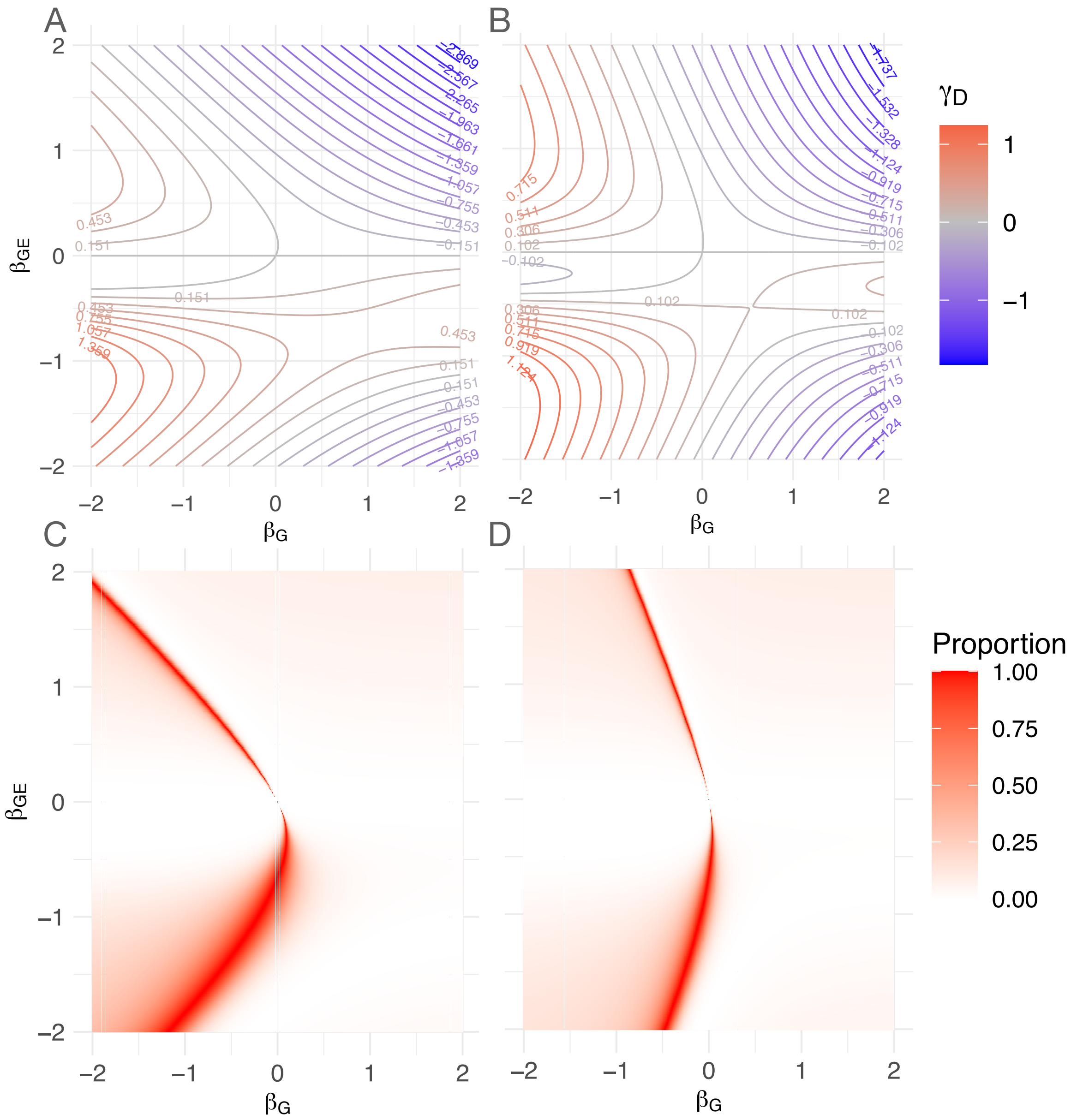

Figure S1: (A-B) show contours of γ_D and (C-D) show heat-maps of the non-additive proportion of genetic variation R_D^2, at different β_G and β_{GE}. The parameter $\beta_E = 0.5$ and $E \sim N(0, 1)$. The MAF is set to $p = 0.3$ in (C-D). The underlying model is assumed to be probit. The prevalence of the binary trait Y is 0.1 on the left column and 0.3 on the right column.
