# Supplementary material for: Detecting latent interaction effects when analyzing binary traits
Source: PLoS Genet. 2025 Aug 22;21(8):e1011822. doi: 10.1371/journal.pgen.1011822 (PMC12396767; doi:10.1371/journal.pgen.1011822)
Supplement: S3 Fig — The individuals (n=100,000) are simulated from a logistic regression model with β0=−1. The latent variable E follows N(0,1) independent of the SNP. (PDF) [file pgen.1011822.s005.pdf]

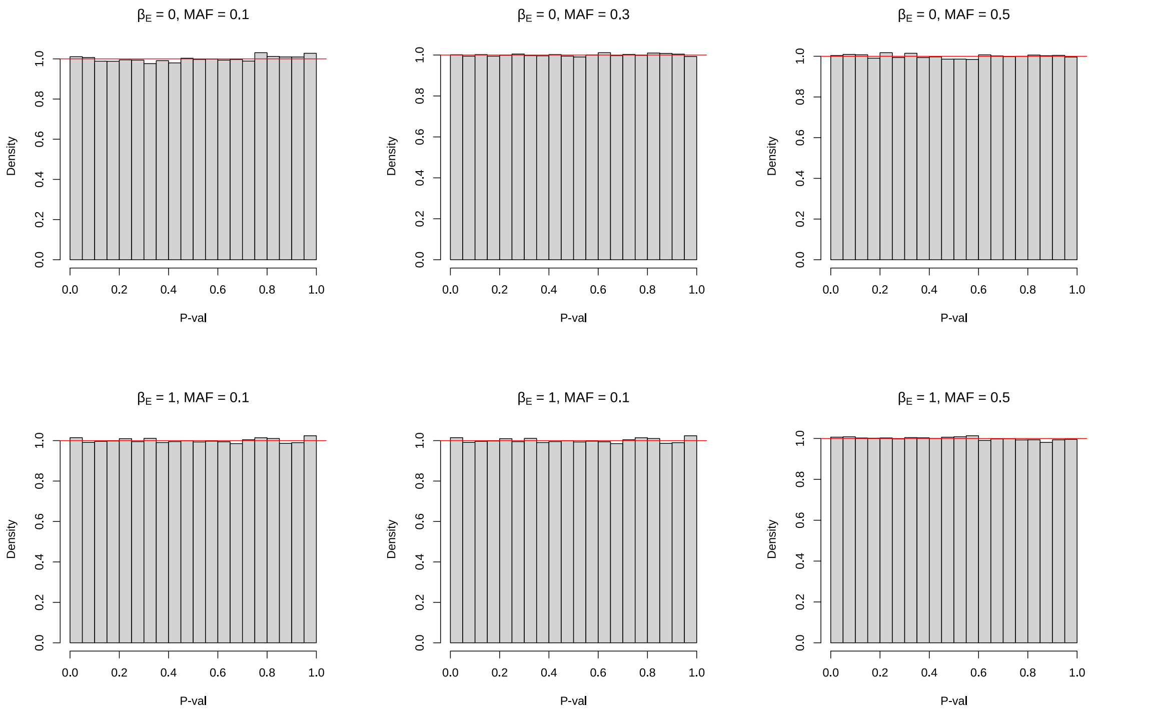

Figure S3: The histograms for the p-values of the proposed joint test when $\beta_{GE} = \beta_G = 0$, at different settings of the MAF and β_E. The individuals ($n = 100,000$) are simulated from a logistic regression model with $\beta_0 = -1$. The latent variable E follows $N(0,1)$ independent of the SNP.
