# Supplementary material for: Detecting latent interaction effects when analyzing binary traits
Source: PLoS Genet. 2025 Aug 22;21(8):e1011822. doi: 10.1371/journal.pgen.1011822 (PMC12396767; doi:10.1371/journal.pgen.1011822)
Supplement: S4 Fig — The binary trait (high cholesterol) has been permuted before the GWAS. (PDF) [file pgen.1011822.s006.pdf]

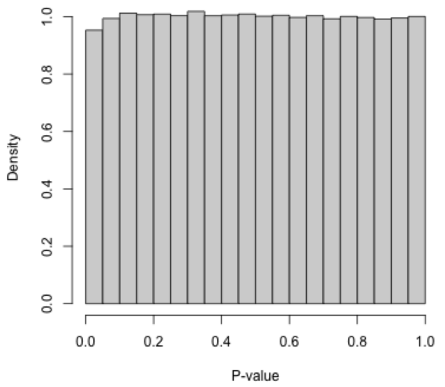


(a) Permuted (indirect) GWAS: Histogram

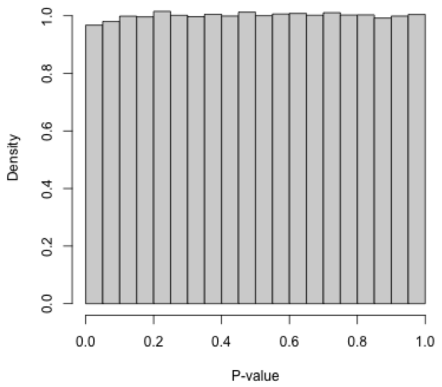


(b) Permuted (joint) GWAS: Histogram

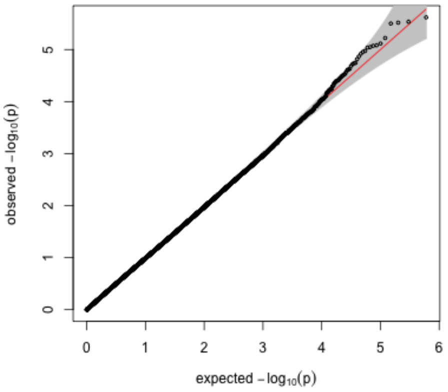


(c) Permuted (indirect) GWAS: QQ-Plot

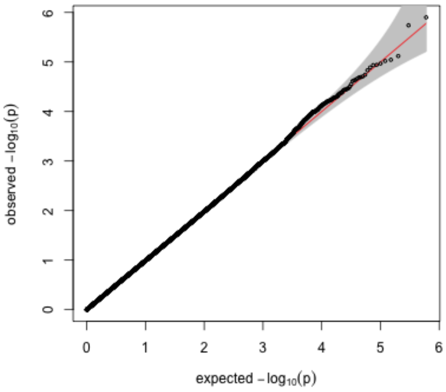


(d) Permuted (joint) GWAS: QQ-Plot

Figure S4: The histograms (a-b) and QQ-plots (c-d) for the GWAS p-values (indirect in the left, joint in the right), for the European population. The binary trait (high cholesterol) has been permuted before the GWAS.
