# Supplementary material for: Detecting latent interaction effects when analyzing binary traits
Source: PLoS Genet. 2025 Aug 22;21(8):e1011822. doi: 10.1371/journal.pgen.1011822 (PMC12396767; doi:10.1371/journal.pgen.1011822)
Supplement: S7 Fig — The size of βE is respectively set to −0.5 (left), 0 (center) and 0.5 (right). The sample size is n=800,000. (PDF) [file pgen.1011822.s009.pdf]

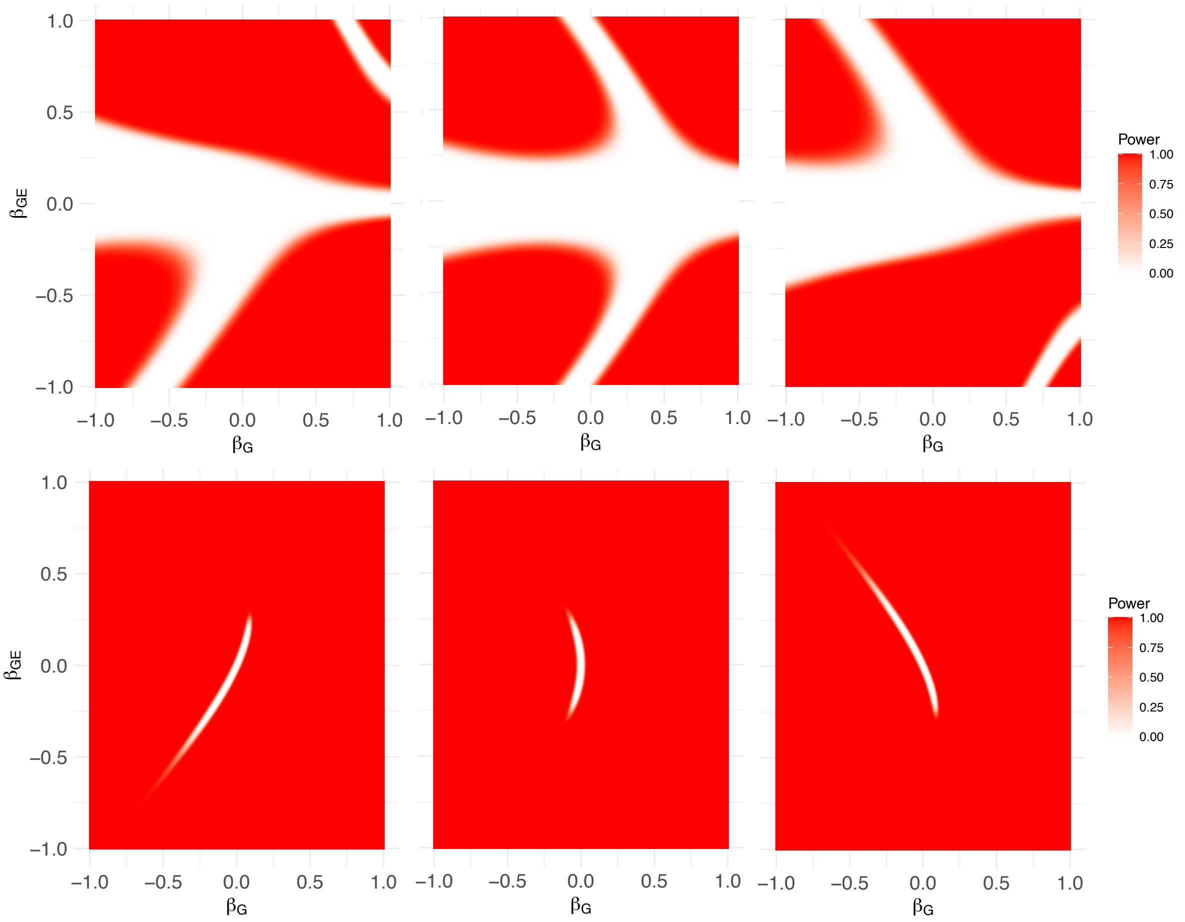

Figure S7: Power of the proposed tests: The power for the proposed indirect test of β_{GE} based on the non-additive effect β_D is shown in the first row, and the power for the proposed joint test of β_{GE} and β_G is shown in the second row. The size of β_E is respectively set to -0.5 (left), 0 (center) and 0.5 (right). The sample size is $n = 800,000$.
