# Supplementary material for: Detecting latent interaction effects when analyzing binary traits
Source: PLoS Genet. 2025 Aug 22;21(8):e1011822. doi: 10.1371/journal.pgen.1011822 (PMC12396767; doi:10.1371/journal.pgen.1011822)
Supplement: Attachment — Submitted filename: response.pdf [file pgen.1011822.s012.pdf]

Response to comments from Reviewer #1

"There is a large body of literature showing that when a genetic variant is involved in an interaction effect on a given continuous outcome, it is expected to be associated with the variance of that outcome. Based on this observation, various approaches have been proposed to pre-select candidates for interaction effect testing based on variance or other scale metrics. This paper proposes an equivalent of that approach for the case of binary outcomes. I actually briefly looked at this question a few years ago but didn't found any solution, so I would like to commend the author for figuring out the proposed approach. Although the path toward the solution is not trivial, the final model is very simple and only consists of testing the significance of a dominance term (on top of the standard additive one). I ran a few toy simulations and qualitatively confirmed the results from the authors: that is, the presence of a dominance effect when a GxE interaction effect is added to the generative model. Overall, the manuscript reads well and I do not have any major concerns."

Response: We sincerely thank the reviewer's appreciation of our work, as well as the kind encouragement. We have carefully addressed each of the comments and suggestions, as detailed in the following point-by-point reply.

Minor comments:

1. "As mentioned above, the proposed solution is very simple in practice: adding a dominance term on top of the additive one. It took me multiple reading and running simulations to make sure I got this right. This should be clearly stated along the manuscript (abstract, intro and conclusion)."

Response: We would like to thank the referee for this helpful suggestion. To further emphasize the practical simplicity of our proposed approach, we have added the following content to the revised manuscript:

- (Page 1, Line 22-27): *"In this paper, we propose an approach to indirectly test interaction effects for binary traits and subsequently propose a joint test that accounts for the main and interaction effects of each SNP during GWAS. The final method is straightforward to implement in practice—it simply involves adding a non-additive (i.e., dominance) term to standard GWAS additive models for binary traits and testing its significance."*
- (Page 4, Line 79-82): *"As a result, the proposed method can be smoothly integrated into existing GWAS pipelines such as PLINK2 (Chang et al., 2015) for binary traits by simply including a non-additive term and testing its effect."*
- (Page 18, Line 387-391): *"The proposed method requires only the addition of a non-additive (dominance) term to the conventional additive regression model and can be implemented directly using the PLINK2 GWAS software (Chang et al., 2015). For other*

commonly used GWAS software, integrating the proposed methodology may involve some additional coding, but the overall implementation should be manageable."

We believe these revisions more clearly emphasize the practical utility and core idea of our proposed framework.

2. " I found Figure 1 and 2 pretty hard to read. I understand that they show the big picture, but given the shape of the curves I am not sure what are the take-home messages. There are simpler plots that would better convey the message found in the main text. For example, for Fig.2, the author state that "the proposed non-additive and joint tests tend to have higher power when betaGE and betaE have opposite signs". A plot showing the power of the tests on the Y axis, and betaGE on X axis would be easier to read. The authors may consider moving those two figures to supp and replace them with plots illustrating the specific characteristics they would like to highlight."

Response: We would like to thank the referee for this helpful suggestion. In the revised manuscript, the original Figures 1 and 2 have been moved to the Supplementary Materials. Following the reviewer's recommendation, we have added a new set of figures (Figures 1-2, Page 26-27) that more directly illustrate the key points described in the main text. Specifically, these new plots show the size of the dominance term γ_D, the dominance proportion of genetic variation R^2_D , the power of the indirect test, and the power of the joint test on the y-axis, with β_{GE} on the x-axis.

3. "Comparing Figure S8 and Figure 3a, there is something that I do not understand. The p-values from the joint test (Figure 3a) appear to be substantially lower than for the marginal (Figure S8) for most of the peaks. For example on chromosome 1, the lowest P for the additive model is ~1e-40, when in comparison it is ~1e-80 for the joint test. The min(P) for the dominance test on that chromosome is 1e-5. From my experience, the joint test is expected to be fairly close to the marginal one, especially if the added term (here the dominance) has a modest effect. I ran multiple simulations but couldn't produce a single scenario where the difference was so large. Some explanations are welcome."

Response: We would like to thank the referee for this insightful question. Along with our revision, we have included an R script that demonstrates a scenario (logistic regression with n = 300,000, $\gamma_0=3$, $\gamma_1=\gamma_2=4.5$) where the joint test yields a much more significant result than the two marginal tests. For each replication, we fixed a given value of the minor allele frequency (MAF), independently simulated n data points from the model described above, and fit a logistic regression model with G_A and G_D (using both the PLINK2 encoding and the orthogonal encoding). We then obtained the p-values for the additive test, dominance test, and joint test based on the Wald statistics from the fitted model. The output is summarized below in Figure R1(a-b) for the convenience of the reviewer.

The key explanation for this phenomenon lies in the correlation between the additive (G_A) and dominance (G_D) terms. In the UKB application, we used the software PLINK2, which uses a non-orthogonal encoding for G_A and G_D. As the MAF decreases, the correlation between these two terms increases. When G_A and G_D are highly correlated, the model can detect that at least one of the terms explains a substantial portion of the variation in the outcome, but can't clearly separate their individual contributions, which is also a common challenge in fine mapping problems (see Wang et al., 2020, for further discussion on this issue). This can result in a highly significant joint test despite relatively modest individual test statistics. On the other hand, if we use orthogonal encodings for G_A and G_D, we agree with the reviewer that the scenario described above would not occur. For comparison, we also repeated the above simulation using orthogonal encoding, and included the results in Figure R1(c–d).

We now briefly discuss this point in the revised manuscript:

- (Page 16, Line 339-344): "*The computation in this example was carried out using the software PLINK2. In PLINK2 (Chang et al., 2015), the non-additive term* G_D *is encoded using an over-dominance scheme (0, 1, 0 for genotypes aa, Aa, AA). Unlike the orthogonal encoding described earlier, this coding does not ensure that* G_A *and* G_D *are uncorrelated. While this non-orthogonality may affect the marginal test for* β_A*, it does not affect the results of the joint test (*$\beta_A = \beta_D = 0$*) or the test of the non-additive effect (*$\beta_D = 0$*).*"

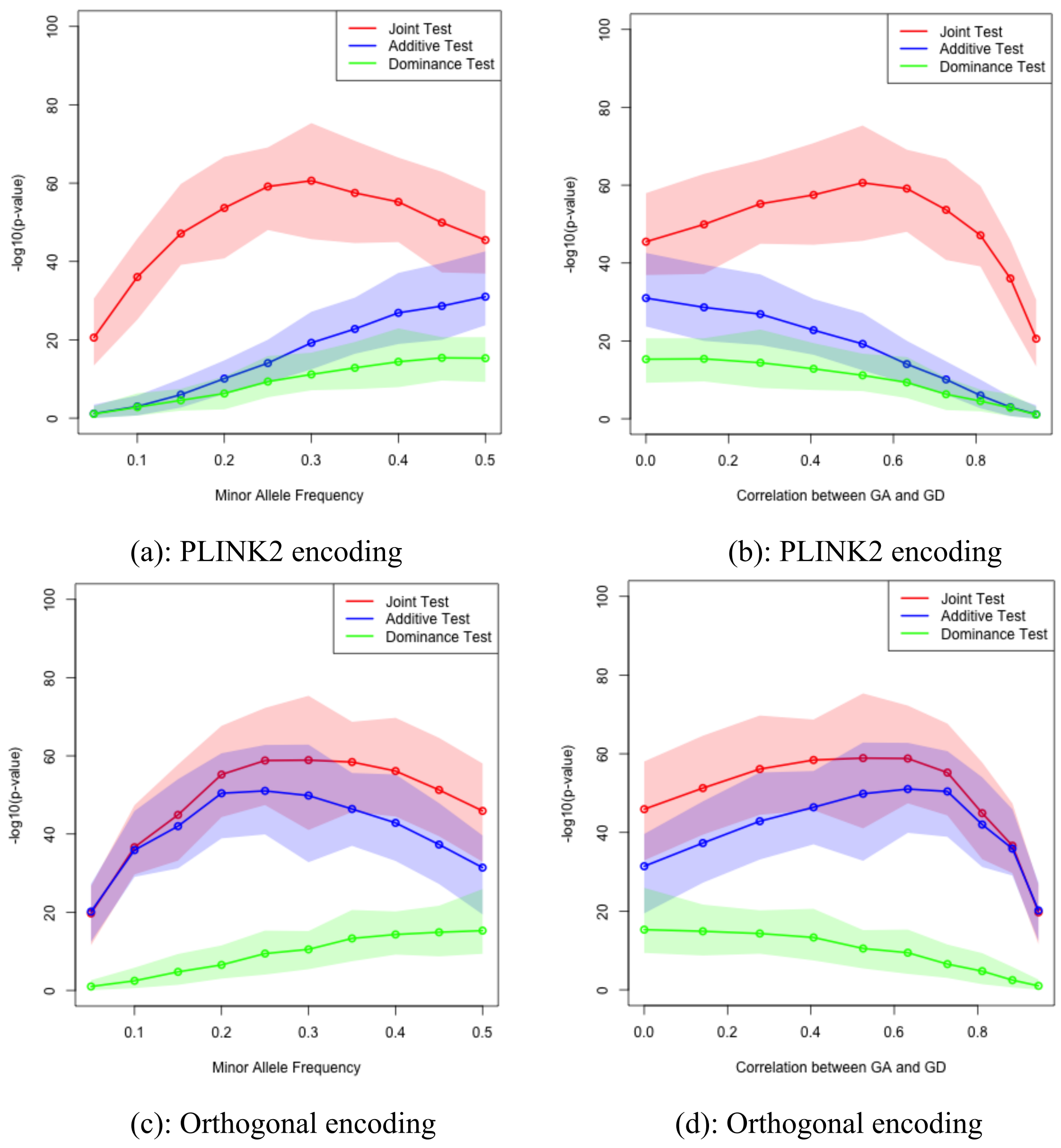

(a): PLINK2 encoding

(b): PLINK2 encoding

(c): Orthogonal encoding

(d): Orthogonal encoding

Figure R1: *The (-log10) p-values from the 2 df joint test, as well as from the 1 df marginal tests for the additive and dominance effects, plotted as a function of minor allele frequency (left), or the correlation between* G_A *and* G_D *(right). Results are based on 50 independent simulations; the mean p-value across replicates is shown as a dotted line, and the 95% interval is represented by the shaded region. For each replication, the binary trait is simulated from a logistic regression with* $\gamma_0=3$, $\gamma_1=\gamma_2=4.5$. G_A *and* G_D *are encoded using PLINK2 encoding on the top two panels and orthogonal coding on the bottom two panels. Full simulation details are provided in the accompanying R scripts (available at the [online code repository]).*

4. "BTW. The authors may consider moving Figure S8 along the other Manhattan in Figure 3 to allow for a direct comparison of the proposed test with the standard additive logistic model."

Response: We would like to thank the referee for this helpful suggestion, and we have followed the suggestion to move the Figure S8 into the main Figure 3 in the revision.

5. "In Figure 3: it seems that plotting the interaction test against the indirect test against each other (-log10(Pinteraction) = f(-log10(Pindirect))) would be more informative if the goal is to highlight the link between the two approaches."

Response: As the reviewer suggested, we have now included a Figure 4(c) that plots the p-values from the two tests.

6. "There are 10 SNPs in Fig 4, but 11 discussed in the earlier part of the results. What happened to the last one? "

Response: In this application, we discussed two distinct sets of SNPs out of the 65 selected SNPs. The first set consists of 11 SNPs identified as having significant interaction effects with rs7412. The second set consists of 10 SNPs identified as having significant non-additive (dominance) effects when interaction with rs7412 is not accounted for.

Figure 4 focuses on the second set of SNPs. Our aim is to follow up on these SNPs to determine whether their non-additive effects can be partially or fully explained by incorporating the interaction with rs7412.

To clarify this distinction, we have added the following explanation to the revised manuscript:

- (Page 17, Line 372-376): "*To confirm that accounting for interaction with rs7412 can indeed explain part of the non-additive (i.e., dominance) effects flagged earlier by the proposed indirect test, we re-applied the test to the 65 selected SNPs, both with and without including the interaction term with rs7412. When the interaction is not considered, we found 10 SNPs with p-values from the non-additive test less than 0.05, and 3 SNPs with p-values less than 5e-8.*"

7. "Introduction: "However, a direct, exhaustive GxG search may be undesirable […] multiple hypothesis testing". May be worth mentioning the computational burden as well!"

Response: We thank the reviewer for this helpful comment. We have revised the sentence accordingly:

- (Page 3, Line 54-56): "*However, a direct, exhaustive GxG search may be undesirable in the genome-wide association study (GWAS) setting because of the large-scale multiple hypothesis testing and the substantial computational burden involved.*"

8. "Introduction: Typo "have also been reported [by] Singhal et al. (2023)."

Response: We thank the referee for catching this typo. We have corrected it in the revised manuscript.

9. "There are parentheses missing in second and third lines of equation (8)"

Response: We appreciate the reviewer for pointing this out. We have revised the formatting of Equation [8] accordingly to improve clarity.

10. "Please provide a reference for the "Fisher orthogonal re-parametrization". I personally have never heard about it before."

Response: The term "*Fisher orthogonal re-parametrization*" was borrowed from Palmer et al., (2023), which referred to the work by Fisher (1919). We have included both references in the revised manuscript as clarification.

<u>**Response to comments from Reviewer #2**</u>

<u>***Comments from the secondary reviewer:***</u>

1. "I have consulted with a biostatistician colleague who has attested to the validity of the statistical methods. This secondary reviewer's comments are the following:

- ***'The proposed model employs a latent trait parameterization to analyze binary traits and detect non-additive gene-environment interactions. The statistical formulation is rigorous and well-justified. While the manuscript states that the choice of test does not affect asymptotic validity, I wonder whether a score test might offer improved finite-sample performance and computational efficiency compared to the Wald test currently implemented.'"***

Response:
To clarify, we chose to use the Wald test in this work in order to align with current practices in the literature and to ensure compatibility with widely used software. We have now emphasized this point more clearly in the revised manuscript:

- (Page 11, Line 219-225): "*The choice of test does not affect the validity of the proposed method, and given the large sample sizes typical in GWAS settings, these tests are expected to have comparable power. The Wald test is often used in GWAS practice because fitting the model to perform the test also yields maximum likelihood estimates and standard errors for each covariate, enabling straightforward reporting of effect sizes and p-values. Therefore, we adopt the Wald test to maintain consistency with existing literature (Yu et al., 2015) and widely used software implementations such as PLINK2 (Chang et al., 2015).*"

We agree that the score test may offer improved computational efficiency, particularly in simpler models without covariates, since it avoids fitting the full model under the alternative hypothesis. We have added the following clarification to the revised manuscript:

- (Page 11, Line 225-228): *"We note, however, that the method presented in this work is also compatible with other types of tests. For example, when only a p-value is needed from a model without additional covariates, a score test can be used to improve computational efficiency."*

Regarding finite-sample performance, we appreciate the reviewer's point that the Wald and score tests may differ slightly in practice. However, given the large sample size in our UK Biobank application, such differences are expected to be minimal. As a practical demonstration, we compared the p-values from the Wald and score versions of the proposed indirect test for the 65 selected SNPs in Section 4. As shown in Figure R2 below, the results are highly consistent.

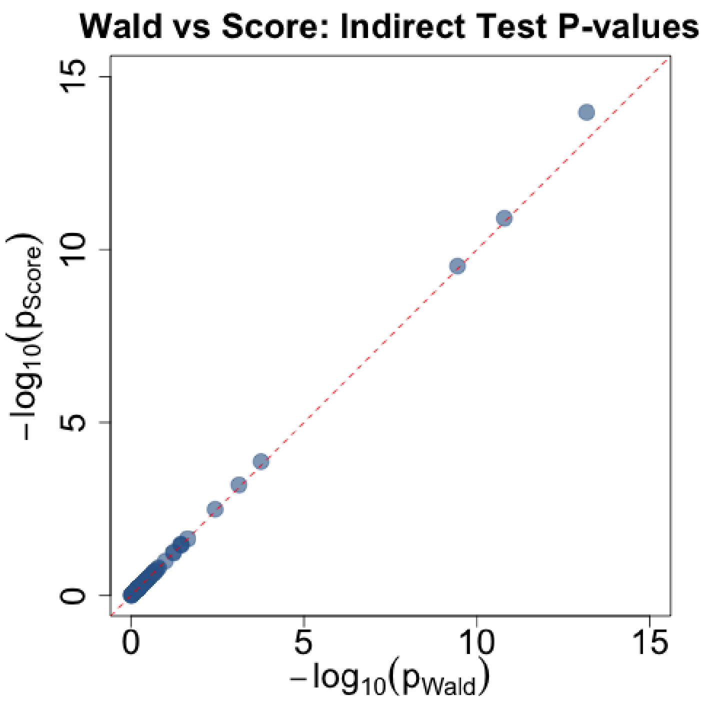

Figure R2: *Comparing the (-log10) p-values from the indirect (non-additive) test for the 65 selected SNPs in Section 4, using either the Wald test (x-axis) or the score test (y-axis). The red dashed line represents the identity line (y=x), indicating agreement between the two tests.*

<u>Comments from the primary reviewer:</u>

1. "While the authors attribute these results to within-locus GxG effects, they do not address the possibility that these results could be explained by a dominance effect at this locus. In fact, a recent analysis of dominance effects in the UK Biobank, which the authors do cite in this manuscript, identified a putative dominant genetic effect of rs7412 in the APOE locus on self-reported high cholesterol (Palmer et al., Science, 2023, PMID: 36996212, Table S4), using the same exact phenotype in the same dataset as those analyzed in this manuscript. This observation raises the concern that the proposed method is simply a recapitulation of previously existing methods and is unable to distinguish between dominance, GxE, and GxG."

Response:

We thank the reviewer for this insightful comment. We agree that indirect tests, whether the proposed method based on the dominance effect for binary traits or existing methods based on variance heteroskedasticity for continuous traits (e.g., Soave et al., 2015; Miao et al., 2022), should be viewed primarily as screening tools. While such methods can highlight candidate loci

for follow-up interaction analysis, they cannot definitively determine whether the observed effects are due to true interaction (GxE or GxG) or are intrinsic to the locus itself.

We have further clarified on this aspect in the following paragraph in our revision:

- (Page 18, Line 408-417) *"Our approach has a similar nature to the approach of Soave et al. (2015) for quantitative traits, where Soave et al. (2015) accounts for latent interactions by jointly testing the genetic effects in the location and the scale of the quantitative trait, and our approach accounts for latent interactions by jointly testing the additive and non-additive genetic effects for the binary trait. In Soave et al. (2015), it is emphasized that we cannot conclude whether the heteroskedasticity is caused by the SNP itself, or by a latent interaction. Similarly for our approach, a significant non-additive effect could be either due to the biological mechanism of the SNP itself, or its interaction with latent variables. These joint tests are valuable for identifying SNPs for more detailed interaction analysis, but their results should not be over-interpreted."*

In our UK Biobank application, our goal is to provide a proof of concept to demonstrate that the proposed indirect approach can effectively prioritize candidates for interaction testing. As shown in our revised manuscript (Figure 4c), the significance from the indirect screening based on dominance effects is predictive of the significance from the GxG test. For the reviewer's convenience, this Figure is also reproduced below as Figure R3.

To further assess whether the dominance effects identified by the indirect test are driven by latent GxG interaction, we also included the following analysis:

- (Page 17, Line 372-376) *"To confirm that accounting for interaction with rs7412 can indeed explain part of the non-additive (i.e., dominance) effects flagged earlier by the proposed indirect test, we re-applied the test to the 65 selected SNPs, both with and without including the interaction term with rs7412. When the interaction is not considered, we found 10 SNPs with p-values from the non-additive test less than 0.05, and 3 SNPs with p-values less than 5e-8."*

These results support the notion that the dominance effect detected near rs7412 – also reported by Palmer et al. (2023) – can at least in part be attributed to the latent interaction.

We believe the revised manuscript now more explicitly communicates that: (1) dominance effects identified via the proposed method in the example may indeed reflect latent interactions, and (2) the method is intended as a screening strategy for follow-up interaction testing, rather than a replacement for direct interaction modeling.

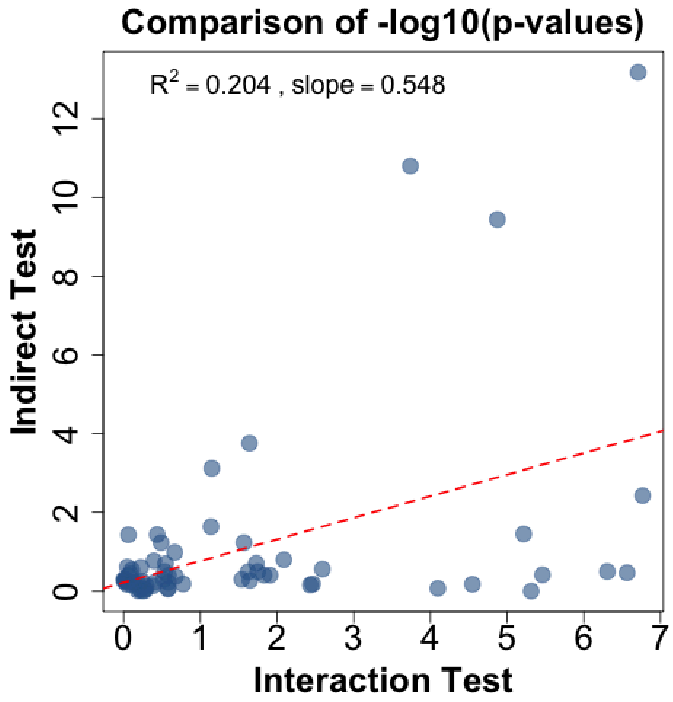

Figure R3: *(Figure 4.C in the revised manuscript) Scatterplot of (-log10) p-values from the indirect (non-additive) test versus the interaction test, for the 65 selected SNPs near rs7412. The red dashed line shows the fitted regression line, with a slope of 0.548 and* R^2 *of 0.204.*

2. "The manuscript could possibly be strengthened by providing an additional application of the method that attempts to replicate an example of GxE identified in previously published literature. This would lend additional relevance to the method and serve as external validation. A possible UK Biobank GxE finding to directly replicate can be found in a Biological Psychiatry article from 2021 by Ye and colleagues (PMID: 33500177, SNP: rs162553, exposure: Townsend deprivation index, outcome: self-harm).
In summary, I believe the general framework laid out in this manuscript is of importance and broad interest to genetics researchers, and the methodology is rigorous. However, substantial additional analysis would be required to demonstrate the practical utility of this approach, the interpretability and biological relevance of its findings, and the overall novelty of the approach."

Response:
We thank the reviewer for this thoughtful comment. Following the suggestion, we attempted to apply the proposed indirect test to the example reported in Ye et al. (2021) and contacted the authors for additional details. In their original analysis, the authors split the full dataset into a discovery and a replication cohort, and reported that the SNP rs162553 was significant in both. As we were unable to reproduce the exact data split used in their analysis, we instead analyzed the full European sample from the UK Biobank. In this full-sample analysis, we did not observe

a significant interaction between rs162553 and the Townsend Deprivation Index ($p = 0.976$). Moreover, the main effect of rs162553 was also not significant ($p = 0.783$). Since the original paper reported only p-values and did not provide the estimated interaction effects in each subset, it is possible that the direction of the interaction effects differed between the discovery and replication cohorts. We contacted the authors for clarification but did not receive a response regarding whether the interaction effects were directionally consistent. To the best of our knowledge, this interaction has not been independently replicated. For these reasons, we decided not to include this example in our manuscript.

While we fully agree that it would be valuable to demonstrate the practical utility of the proposed method through the discovery of novel GxE interactions, conducting a comprehensive genome-wide search is a substantial undertaking that falls outside the scope of the current manuscript. Rather, the main goal of this work is to (i) demonstrate that an indirect test for interaction is theoretically possible for binary traits, and (ii) provide a practical interpretation for non-additive effects identified in binary trait GWAS, as illustrated through the UKB example presented in our manuscript. To make these goals more explicit, we have added the following clarification to the revised manuscript:

- (Page 17, Line 382-387): *"Using heteroskedasticity to indirectly test for a latent interaction is well-established in the analysis of quantitative traits, and has led to many scientific insights over the human genome. However, none of the existing approaches of indirect testing could be applied when the trait of interest is binary. In this paper, we (i) derive, for the first time, an indirect test for binary traits, and in doing so, (ii) offer a practical interpretation for non-additive effects identified in binary trait GWAS."*

We hope the revised text now more clearly highlights both the methodological contribution and the interpretive value of our approach.

3. "Finally, while the authors provide detailed power analyses, the likelihood of identifying meaningful results with current GWAS sample sizes is unclear. The UK Biobank application only flagged a single locus, APOE, whose epistatic effects have already been widely studied in Alzheimer's disease and metabolic traits, raising the concern that the proposed method may only be powered to detect latent interactions at loci with very large effect sizes."

Response:

We thank the reviewer for raising this point. We acknowledge that our original figures for illustrating power may not have been sufficiently clear. In response to this comment (along with the second minor comment from Reviewer #1), we have re-designed Figure 2 to more effectively demonstrate how the power of the proposed indirect test varies with the magnitudes of the main and interaction effects. This illustration assumes a sample size of 300,000, which closely approximates the actual sample size of 276,658 in our UK Biobank application.

In general, the power of the indirect test is influenced by a complex interplay of factors, including the effect sizes of β_G, β_E and β_{GE}, as well as the trait prevalence, the minor allele frequency, and the variance of the latent variable E. These dependencies are captured implicitly through the transformation described in Equation (13). As a result, a large effect size of β_{GE} or β_G is not always required for the indirect test to achieve reasonable power. Conversely, increasing the size of β_{GE} does not always guarantee higher power due to the non-linear dependencies involved.

The revised Figure 2(a–b) is included as Figure R4 below for the reviewer's convenience.

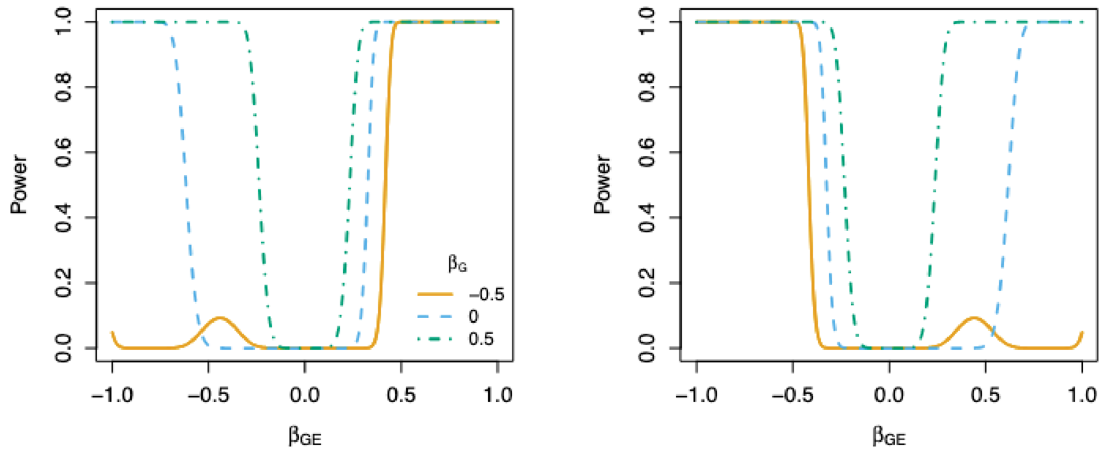

(a) Power (indirect) when $\beta_E = -0.5$ (b) Power (indirect) when $\beta_E = 0.5$

Figure R4: *(Figure 2.a-b in the revised manuscript) The power for the proposed non-additive test based on γ_D (1 df). The size of β_E is respectively set to -0.5 (left) and 0.5 (right). The sample size is n = 300,000.*

<u>Additional comments:</u>

1. "As mentioned above, the authors should clearly state that they have implemented their method using an existing Plink function, rather than developing new software"

Response:
We thank the reviewer for this helpful suggestion. Indeed, one practical strength of the proposed method is that it can be readily implemented using existing software. To make this point more explicit, we have added the following clarification in the revised manuscript:

- (Page 18, Line 387-391): *"The proposed method requires only the addition of a non-additive (dominance) term to the conventional additive regression model and can be implemented directly using the PLINK2 GWAS software (Chang et al., 2015). For other*

commonly used GWAS software, integrating the proposed methodology may involve some additional coding, but the overall implementation should be manageable."

In addition, we now clarify that the UKB application was conducted using PLINK2:

- (Page 16, Line 339):*"The computation in this example was carried out using the software PLINK2 (Chang et al., 2015)."*

2. "The manuscript title contains "gene-environment interaction," and the manuscript seems to focus mainly on GxE, yet the introduction alternates between discussing GxE and GxG. Further, the UK Biobank application, which is the only application of the method provided in the manuscript that uses real data, identifies an example of GxG. However, there is no example application of the method that identifies GxE or seeks to replicate a previously discovered GxE. I would suggest reframing the title, introduction, and discussion to more broadly refer to GxE and GxG, to make it clear that the method applies to both concepts. Further, as noted above, the authors should clarify that the method cannot distinguish between GxE, GxG, and dominance effects."

Response:
We thank the reviewer for this suggestion. In response, we have reframed our title to ***"Detecting latent interaction effects when analyzing binary traits"***. We have also updated the abstract and introduction to emphasize that the proposed method is applicable to any form of latent interaction—whether gene–environment (GxE) or gene–gene (GxG)—and is not restricted to one specific type.

Additionally, we have clarified that, analogous to other indirect methods (e.g., Soave et al., 2015), our approach cannot distinguish between true biological dominance and latent interaction effects. We now make this explicit in the revised manuscript with the following text:

- (Page 18, Line 412-417): *"In Soave et al. (2015), it is emphasized that we cannot conclude whether the heteroskedasticity is caused by the SNP itself, or by a latent interaction. Similarly for our approach, a significant non-additive effect could be either due to the biological mechanism of the SNP itself, or its interaction with latent variables. These joint tests are valuable for identifying SNPs for more detailed interaction analysis, but their results should not be over-interpreted."*

3. "Please describe your methodology for the simulations generated under the theoretical null hypothesis that were used to assess type I error rate in Table 1."

Response:
In response, we have expanded that section to provide detailed descriptions of how the data were simulated under the theoretical null hypothesis and how the p-values were computed:

- (Page 13, Line 259-267): *"To assess the type I error rate of the proposed non-additive test that detects the latent GxE effect β_{GE} based on the non-additive effect β^*_D, we simulate n = 100,000 independent individuals under the theoretical null hypothesis that $\beta_{GE} = 0$, using the logistic model in Equation [6]. We fix $\beta_0 = -1$ and $\beta_G = 0.5$, and independently simulate E ~ N(0,1) and G under the HWE. We consider six settings defined by the combinations of minor allele frequency (MAF) of G in {0.1, 0.3, 0.5} and β_E in {0, 1}. The prevalence of the trait varies roughly between 0.3 and 0.4 across these six settings. For each of the six settings, we perform B = 100,000 independent replications and compute one p-value per replication using the Wald test statistic in Equation [18], resulting in B independent p-values."*

4. "For Figure 1 A-B, please explain, intuitively, why γD takes on negative values for most parameter settings, including when βG and βGE are both positive values. How is the sign of γD relevant to its interpretation? Similarly, for Figure 1 C-D, please explain why the proportion of genetic variation explained by the non-additive component is dependent on the sign of the SNP effect βG."

Response:
As suggested by the reviewer, we have added the following explanation to our revised manuscript to clarify that the interpretation of both γ_D and R^2_D in relation to specific regression parameters is inherently difficult, due to multiple non-linear function compositions in the binary outcome model:

- (Page 9, Line 198-206): *"The non-additive effect γ_D represents the deviation from linearity in the genotype-dependent component of the linear predictor, specifically capturing the extent to which the value for the heterozygous genotype (G = 1) deviates from the midpoint between the two homozygous genotypes (G = 0 and G = 2). A negative γ_D indicates a form of sub-additivity, where the effect of carrying two copies of the risk allele is disproportionately smaller than expected under an additive model. Notably, such a pattern can emerge even when both β_G and β_{GE} are positive, due to the non-linear composition of functions in Equation [11] that define the binary outcome model (as illustrated in Figure [1](a-b))."*
- (Page 10, Line 206-208): *"Note that Figure [1](b) is simply a mirror image of Figure [1](a) with respect to β_{GE}, because γ_D remains unchanged when both β_E and β_{GE} are replaced by $-\beta_E$ and $-\beta_{GE}$, as shown in Equation [13]."*
- (Page 11, Line 213-216): *"Similar to γ_D, the proportion of variance explained by the non-additive component depends in a highly non-linear manner on all of the regression parameters, due to the complex functional composition in Equation [11] and the non-linear link function."*

5. "Figure 2: The figure legend indicates that the first row represents power estimates for the non-additive test for a sample size of N=300,000, while the second row represents power estimates for the joint test for a sample size of N=30,000. Why did you choose different sample sizes for the two tests? Also, if the sample size for the tests in the first row is 10x greater than in the second row, why is the power substantially lower across the parameter space in the first row?"

Response:
We acknowledge that our original version of Figure 2 may have caused confusion. In response, we have redesigned the figure as described in our response to Minor Comment #2 from Reviewer #1.

The different sample sizes in the two rows were chosen deliberately to better illustrate the behavior of each test. If we had used the same large sample size (e.g. n = 300,000) for both tests, the joint test (shown in the second row) would have had near-complete power across much of the parameter space, obscuring differences in power across different settings. Using a smaller sample size for the joint test allows us to visualize how power changes under varying parameter values more clearly. This also explains why the power in the first row appears lower despite a larger sample size: the non-additive test (top row) is based on a 1-degree-of-freedom test, while the joint test (bottom row) is based on a 2-degree-of-freedom test. The tests target different hypotheses and are not directly comparable in power even under the same sample size.

To clarify this in the manuscript, we have revised the figure legend as follows:

- (Figure 2 Caption, Page 27): *"Power of the proposed tests: The power for the proposed non-additive test (1 df) based on γ_D is shown in the first row, and the power for the proposed joint test (2 df) of β_{GE} and β_G is shown in the second row. The size of β_E is respectively set to -0.5 (left) and 0.5 (right). The sample sizes are set to n=300,000 for the non-additive test and n=30,000 for the joint test, in order to better illustrate their power behavior under different parameter settings. The significance level is set to $\alpha = 5 \times 10^{-8}$."*

<u>Minor edits:</u>

1. *Parts of the results section reads like a methods section. The authors may consider revising.*
2. *The discussion states that hypertension was studied in the UK Biobank analysis (this should be cholesterol).*
3. *Some panels in Figure S5 seem to be identical to those in Figure 2.*
4. *Figure S4 is referenced in the text after Figures S5-S7.*
5. *Tables S1 and S2 do not appear to be referenced in the main text.*
6. *Figure S8 is cut off at the bottom*

Response: We thank the reviewer for these helpful suggestions and for pointing out several inconsistencies and typographical errors in the original manuscript. We have carefully addressed each of these issues as follows:

1. We have revised and restructured the ***"Results"*** section to improve clarity. Specifically, the type I error and power analyses have been moved to a new section titled ***"Method Verification"***.
2. We have corrected the typo in the ***"Discussion"*** section; the analysis focused on self-reported high cholesterol, not hypertension.
3. We have redesigned Figure 2, as described in our earlier response, to avoid overlap with the supplementary figures and ensure clarity.
4. We have corrected the ordering of supplementary figures so that they are cited in the manuscript in the correct order (i.e., Figure S4 now appears before Figures S5–S7).
5. Table S1 is now referenced at Line 188 (Page 10), and Table S2 is now referenced at Line 363 (Page 17).
6. We have re-plotted Figure S8 (now Figure 3b to fix the issue of the panel being cut off at the bottom.

References

1. Fisher, R. A. (1919). XV.—The correlation between relatives on the supposition of Mendelian inheritance. *Earth and Environmental Science Transactions of the Royal Society of Edinburgh*, 52(2):399–433.
2. Palmer, D. S., Zhou, W., Abbott, L., Wigdor, E. M., Baya, N., Churchhouse, C., Seed, C., Poterba, T., King, D., Kanai, M., et al. (2023). Analysis of genetic dominance in the uk biobank. *Science*, 379(6639):1341–1348.
3. Miao, J., Lin, Y., Wu, Y., Zheng, B., Schmitz, L. L., Fletcher, J. M., & Lu, Q. (2022). A quantile integral linear model to quantify genetic effects on phenotypic variability. *Proceedings of the National Academy of Sciences*, *119*(39), e2212959119.
4. Soave, D., Corvol, H., Panjwani, N., Gong, J., Li, W., Boëlle, P. Y., ... & Sun, L. (2015). A joint location-scale test improves power to detect associated SNPs, gene sets, and pathways. *The American Journal of Human Genetics*, *97*(1), 125-138.
5. Ye, J., Wen, Y., Sun, X., Chu, X., Li, P., Cheng, B., ... & Zhang, F. (2021). Socioeconomic deprivation index is associated with psychiatric disorders: an observational and genome-wide gene-by-environment interaction analysis in the UK Biobank cohort. *Biological psychiatry*, *89*(9), 888-895.
6. Chang, C. C., Chow, C. C., Tellier, L. C., Vattikuti, S., Purcell, S. M., & Lee, J. J. (2015). Second-generation PLINK: rising to the challenge of larger and richer datasets. *Gigascience*, 4(1), s13742-015.
7. Wang, G., Sarkar, A., Carbonetto, P. & Stephens, M. (2020). A simple new approach to variable selection in regression, with application to genetic fine mapping. *Journal of the Royal Statistical Society, Series B* 82.
