# Supplementary material for: Detecting latent interaction effects when analyzing binary traits
Source: PLoS Genet. 2025 Aug 22;21(8):e1011822. doi: 10.1371/journal.pgen.1011822 (PMC12396767; doi:10.1371/journal.pgen.1011822)
Supplement: Attachment — Submitted filename: response_auresp_2.pdf [file pgen.1011822.s013.pdf]

## Response to comments from Reviewer #1

***"Still, one point needs clarification. On line 347-348, the authors state "For comparison, the Manhattan plot from the traditional additive GWAS is also shown in Figure [3](b)." It looks like the authors are referring to the 1df test of beta_A obtained from a PLINK2 run where both G_A and G_D have been modelled (and the R script provided on git appears to agree with this). If so, this is unfair (for the reasons provided by the authors in their response) and the "traditional additive GWAS" should refer to marginal additive effect of the variants where the dominance effect is not modelled at all (i.e. the model Y = G + covariates, where G is coded as 0,1,2). Assuming I am correct, the authors should update Figure 3b and replace it with a standard additive GWAS."***

**Response:**
We would like to thank the referee for this constructive comment. Indeed, our original Figure 3(b) was based on the marginal additive effect ($\beta_A$) estimated from a genotypic model including both additive and dominance terms (i.e., both $G_A$ and $G_D$ were included as covariates). We agree that, to better reflect the standard definition of a traditional additive GWAS, the fitted model should not include the dominance term.

In response, we followed the reviewer's suggestion and re-ran the additive GWAS using a model that excludes the dominance term $G_D$. The Manhattan plot in Figure 3(b) has been updated accordingly. For convenience, the updated figure is also attached in the following page.

To further clarify that the traditional GWAS does not include $G_D$ in the model, we have revised the relevant sentence in the manuscript as follows:

- (Page 16, Line 347-349): *"For completeness, the Manhattan plot from the traditional additive GWAS—where the dominance term $G_D$ is not included in the model—is also shown in Figure 3 (GC $\lambda$ = 1.139)."*

While this update does not alter our main conclusions, we believe it indeed improves the clarity and rigor of the comparison. In addition, we have updated the publicly available Zenodo dataset and GitHub code repository to reflect this change and ensure full reproducibility.

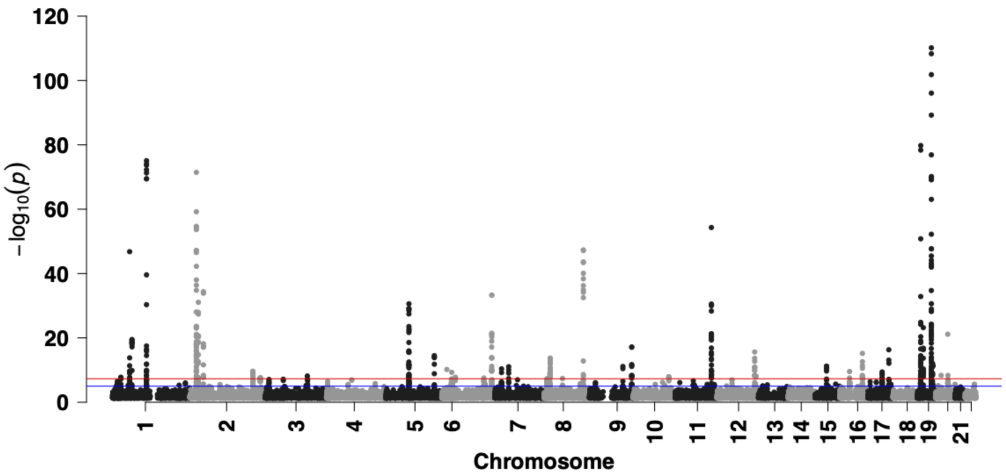


***Figure 3(b):*** *GWAS result using the traditional additive test, where the dominance term is not included in the model.*
