# Supplementary material for: Detecting latent interaction effects when analyzing binary traits
Source: PLoS Genet. 2025 Aug 22;21(8):e1011822. doi: 10.1371/journal.pgen.1011822 (PMC12396767; doi:10.1371/journal.pgen.1011822)
Supplement: Attachment — Submitted filename: PGENETICS-D-24-01489 Reviewer Comments.docx [file pgen.1011822.s011.docx]

**Have all data underlying the figures and results presented in the manuscript been provided?**

Yes

**Comments to the Authors:** (Limit 20,000 Characters)

Please note that I (the primary reviewer) am not a statistician and am unable to evaluate the entirety of the statistical derivations in this manuscript. However, I have consulted with a biostatistician colleague who has attested to the validity of the statistical methods. This secondary reviewer’s comments are the following:

*“The proposed model employs a latent trait parameterization to analyze binary traits and detect non-additive gene-environment interactions. The statistical formulation is rigorous and well-justified. While the manuscript states that the choice of test does not affect asymptotic validity, I wonder whether a score test might offer improved finite-sample performance and computational efficiency compared to the Wald test currently implemented.”*

My (the primary reviewer’s) comments are below:

In this manuscript, Zhang and colleagues describe a statistical test for latent gene-environment (GxE) and gene-gene (GxG) interactions in the analysis of binary traits, arguing that this can essentially be achieved using a traditional dominance test. The analysis of GxE and GxG is broadly relevant for human geneticists, as the evaluation of epistatic interactions as possibly contributing to the “missing heritability” of complex traits is increasingly of interest in the field. The authors describe two tests for evaluating latent GxE and GxG effects on binary traits: a “non-additive” test that tests the departure of the genetic effect from additivity, and a “joint test” to simultaneously test the main genetic effect and the latent interaction effect.

It seems that the authors are arguing that testing for latent GxE or GxG is statistically equivalent to testing for a dominance effect. The script in the provided github repository indicates that the authors have simply run the dominance deviation test using the “--genotypic" command in the Plink2 software for both their “non-additive test” and “joint test.” The authors apply this method by running a GWAS of high cholesterol in the UK Biobank, using this “--genotypic" flag. They find that the only SNPs flagged by both the non-additive and join tests are in the *APOE* locus. The authors note that this finding is not unexpected, given previous literature suggesting GxG effects between nearby SNPs in the *APOE* locus on cholesterol levels. They then directly perform a GxG analysis between the lead flagged SNP (rs7412) and nearby SNPs, finding that the identified latent interaction is largely accounted for by these GxG interactions. However, while the authors attribute these results to within-locus GxG effects, they do not address the possibility that these results could be explained by a dominance effect at this locus. In fact, a recent analysis of dominance effects in the UK Biobank, which the authors do cite in this manuscript, identified a putative dominant genetic effect of rs7412 in the *APOE* locus on self-reported high cholesterol (Palmer et al., *Science*, 2023, PMID: 36996212, Table S4), using the same exact phenotype in the same dataset as those analyzed in this manuscript. This observation raises the concern that the proposed method is simply a recapitulation of previously existing methods and is unable to distinguish between dominance, GxE, and GxG.

The manuscript could possibly be strengthened by providing an additional application of the method that attempts to replicate an example of GxE identified in previously published literature. This would lend additional relevance to the method and serve as external validation. A possible UK Biobank GxE finding to directly replicate can be found in a *Biological Psychiatry* article from 2021 by Ye and colleagues (PMID: 33500177, SNP: rs162553, exposure: Townsend deprivation index, outcome: self-harm). Another option would be to replicate more well-established candidate gene GxE findings from prior literature.

In summary, I believe the general framework laid out in this manuscript is of importance and broad interest to genetics researchers, and the methodology is rigorous. However, substantial additional analysis would be required to demonstrate the practical utility of this approach, the interpretability and biological relevance of its findings, and the overall novelty of the approach. Finally, while the authors provide detailed power analyses, the likelihood of identifying meaningful results with current GWAS sample sizes is unclear. The UK Biobank application only flagged a single locus, *APOE*, whose epistatic effects have already been widely studied in Alzheimer’s disease and metabolic traits, raising the concern that the proposed method may only be powered to detect latent interactions at loci with very large effect sizes.

Additional comments:

1. As mentioned above, the authors should clearly state that they have implemented their method using an existing Plink function, rather than developing new software.
2. The manuscript title contains “gene-environment interaction,” and the manuscript seems to focus mainly on GxE, yet the introduction alternates between discussing GxE and GxG. Further, the UK Biobank application, which is the only application of the method provided in the manuscript that uses real data, identifies an example of GxG. However, there is no example application of the method that identifies GxE or seeks to replicate a previously discovered GxE. I would suggest reframing the title, introduction, and discussion to more broadly refer to GxE and GxG, to make it clear that the method applies to both concepts. Further, as noted above, the authors should clarify that the method cannot distinguish between GxE, GxG, and dominance effects.
3. Please describe your methodology for the simulations generated under the theoretical null hypothesis that were used to assess type I error rate in Table 1.
4. For Figure 1 A-B, please explain, intuitively, why γD takes on negative values for most parameter settings, including when βG and βGE are both positive values. How does the sign of γD relevant to its interpretation? Similarly, for Figure 1 C-D, please explain why the proportion of genetic variation explained by the non-additive component is dependent on the sign of the SNP effect βG.
5. Figure 2: The figure legend indicates that the first row represents power estimates for the non-additive test for a sample size of N=300,000, while the second row represents power estimates for the joint test for a sample size of N=30,000. Why did you choose different sample sizes for the two tests? Also, if the sample size for the tests in the first row is 10x greater than in the second row, why is the power substantially lower across the parameter space in the first row?

Minor edits:

- Parts of the results section reads like a methods section. The authors may consider revising.
- The discussion states that hypertension was studied in the UK Biobank analysis (this should be cholesterol)
- Some panels in Figure S5 seem to be identical to those in Figure 2
- Figure S4 is referenced in the text after Figures S5-S7
- Tables S1 and S2 do not appear to be referenced in the main text
- Figure S8 is cut off at the bottom
